# Characterising Hepatitis D Virus in the UK From 2011 to 2021

**DOI:** 10.1111/jvh.70205

**Published:** 2026-08-09

**Authors:** R. Simmons, S. Ijaz, R. Jackson, K. Jack, R. Mulongeni, R. Raghu, A. Riddell, A. Elsharkawy, A. Uriel, A. Koffas, A. Chaika, A. Lawson, A. Cole, A. McEwan, A. Brown, B. Stone, C. Levick, D. Byrne, D. Forton, D. MacDonald, D. Forde, E. Nastouli, E. Barnes, E. Sumner, E. Page, I. Carey, F. J. Vilar, J. Maggs, J. Collier, J. Shepherd, K. Agarwal, K. Bicknell, K. Ala, K. Dear, L. Ratcliffe, M. Bruce, H. Zhang, L. Rivett, M. Maes, M. Ankcorn, M. Aldersley, M. Prince, M. Lemoine, M. Foxton, M. Wright, M. Cramp, M. Wiselka, N. Easom, P. Richardson, R. Gunson, R. Herbert, R. Blackwell, R. Jelley, R. Aspinall, R. Simpson, R. Clewes, S. Ahmad, S. Mcpherson, S. Ryder, S. Verma, T. Lampejo, V. Sathyanarayana, W. Gelson, D. Leeman, M. Desai, S. Mandal, P. T. Kennedy, W. L. Irving

**Affiliations:** ^1^ UK Health Security Agency London UK; ^2^ Nottingham University Hospitals NHS Trust Nottingham UK; ^3^ Barts Health NHS Trust London UK; ^4^ NIHR Biomedical Research Centre at University Hospitals Birmingham NHS Foundation Trust Birmingham UK; ^5^ University Hospitals Birmingham NHS Foundation Trust Birmingham UK; ^6^ Manchester University NHS Foundation Trust Manchester UK; ^7^ University Hospitals of Derby and Burton NHS Foundation Trust Derby UK; ^8^ Imperial College Healthcare NHS Trust London UK; ^9^ Sheffield Teaching Hospitals NHS Foundation Trust Sheffield UK; ^10^ Royal Devon and Exeter NHS Foundation Trust Exeter UK; ^11^ Liverpool University Hospitals NHS Foundation Trust Liverpool UK; ^12^ St George's University Hospitals NHS Foundation Trust London UK; ^13^ Royal Free London NHS Foundation Trust London UK; ^14^ Public Health Wales Cardiff UK; ^15^ University College London Hospital NHS Foundation Trust London UK; ^16^ Oxford University Hospitals NHS Foundation Trust Oxford UK; ^17^ Nuffield Department of Medicine University of Oxford Oxford UK; ^18^ Micropathology Ltd Coventry UK; ^19^ Leeds Teaching Hospitals NHS Trust Leeds UK; ^20^ Institute of Liver Studies Kings College Hospital London UK; ^21^ Buckinghamshire Healthcare NHS Trust Aylesbury UK; ^22^ The Lothian University Hospitals NHS Trust Edinburgh UK; ^23^ Portsmouth Hospitals University NHS Trust Portsmouth UK; ^24^ Hampshire Hospitals NHS Foundation Trust Basingstoke UK; ^25^ Chesterfield Royal Hospital NHS Foundation Trust Chesterfield UK; ^26^ Cambridge University Hospitals NHS Foundation Trust Cambridge UK; ^27^ University Hospital Southampton NHS Foundation Trust Southampton UK; ^28^ University Hospitals Plymouth NHS Trust Plymouth UK; ^29^ University Hospitals of Leicester NHS Trust Leicester UK; ^30^ Hull University Teaching Hospitals NHS Trust Kingston upon Hull UK; ^31^ West of Scotland Specialist Virology Centre Gartnavel General Hospital Glasgow UK; ^32^ Liver Unit and NIHR Biomedical Research Centre, Newcastle Upon Tyne Hospitals NHS Foundation Trust and Translational & Clinical Research Institute Newcastle University Newcastle upon Tyne UK; ^33^ NIHR Nottingham Biomedical Research Centre at Nottingham University Hospitals NHS Trust Nottingham UK; ^34^ Department of Clinical and Experimental Medicine, Brighton & Sussex Medical School University of Brighton and University of Sussex Brighton UK; ^35^ Department of Gastroenterology and Hepatology (Digestive Diseases), Royal Sussex County Hospital University Hospitals Sussex NHS Foundation Trust Brighton UK; ^36^ Barnsley Hospital NHS Foundation Trust Barnsley UK; ^37^ Queen Mary University of London London UK

## Abstract

Little is known about hepatitis B virus (HBV) and Hepatitis D virus (HDV) coinfection in the UK. HDV screening is recommended among all individuals with HBV, with coinfection associated with increased disease progression. Using data on HDV testing, shared by 11 laboratories in the UK who reported undertaking HDV testing, and national testing data for HBV we established a cohort of individuals tested for HDV, investigating testing pathways, describing characteristics and outcomes of those HDV‐RNA positive to inform targeted interventions for testing, diagnosis and linkage to care. Demographic information, clinical assessments, treatment and recent laboratory results from NHS trusts, and through linkage to national healthcare datasets was collected for HDV‐RNA positive individuals. Between 2011 and 2021, 42% of individuals newly diagnosed with HBV in England were linked to an HDV test. Anti‐HDV positivity was 5.0%, 55.8% were HDV‐RNA tested and 45.7% were ever HDV‐RNA positive. HDV‐RNA positivity in the absence of an anti‐HDV result was 6.4%. Among individuals who were HDV‐RNA positive, 94.3% were non‐UK born, with 24 different primary languages reported, 49% had evidence of treatment, and 48% had evidence of cirrhosis. 10% had died of which 58.4% died of liver disease. In conclusion, our findings indicate that HDV testing in HBV positive individuals in the UK is sub‐optimal against testing guidelines, including RNA testing among those who are anti‐HDV positive. Considerable work is needed to improve the patient care pathway, to ensure patients are appropriately diagnosed, linked and retained in care, with adequate access to treatment.

## 1 Introduction

Individuals coinfected with hepatitis B virus (HBV) and hepatitis D virus (HDV) are at increased risk of liver disease progression and higher rates of severe disease outcomes compared with HBV mono‐infected individuals. A systematic review covering six WHO regions estimated that globally, 5% of individuals living with chronic HBV (CHB) are also living with HDV, ranging from 3.0% in European regions to 6.0% in African regions [1]. HDV infection either occurs as a simultaneous acute infection with HBV, which is likely to result in clearance or as a super‐infection, more likely to result in HDV persistence and accelerated disease progression [2]. Testing guidelines for HDV differ: the European Association for the Study of the Liver (EASL) and the UK National Institute for Health and Care Excellence (NICE) recommend all individuals positive for CHB are screened for HDV [3], whereas the American Association for the Study of Liver Diseases (AASLD) recommends testing for those at high risk of HDV (people living with HIV, people who inject drugs, men who have sex with men, and patients from endemic regions) [4].

Little is known about the characteristics and outcomes of individuals HBV/HDV co‐infected in England. Data from London‐based clinics showed testing coverage for those HBsAg positive was 40% [5], and anti‐HDV prevalence ranged from 2.0% to 8.5% [6, 7]. Recent developments in treatment for HDV include bulevirtide for adults with compensated liver disease with evidence of significant fibrosis, who have not responded to peginterferon alfa‐2a or who cannot have interferon‐based therapy, which, when administered, reduces HDV viraemia levels and normalises liver transaminase levels in a significant number of patients [8]. Establishing a national cohort of individuals tested for HDV is critical for characterising the epidemiology and disease burden of co‐infected patients, as well as for monitoring testing coverage and providing a baseline for auditing against national testing and treatment guidelines.

Using data submitted by laboratories undertaking testing for HDV, clinical data provided by hepatology or infectious diseases clinics, and linkage to national healthcare datasets, we describe testing pathways for individuals diagnosed with CHB in the UK and provide numbers and characteristics of those who are anti‐HDV and/or HDV‐RNA positive.

## 2 Methods

In 2021, a questionnaire was circulated to the UK Clinical Virology Network (CVN) [9], a group of laboratories across all major centres through the UK, asking which laboratories were undertaking HDV testing, and which tests they offered (anti‐HDV and/or HDV‐RNA). HDV RNA assays used are listed in the Section S1. Through the questionnaire we identified 12 laboratories who undertook HDV testing; 11 agreed to share raw testing data directly with UKHSA. These laboratories represent both diagnostic and reference testing.

Testing data on HDV reported between 2011 and 2021 included date of specimen, test result, hospital, and clinical service/setting of test. Data was collated, cleaned, and deduplicated using a combination of name, date of birth, NHS Number, and hospital number, grouping multiple tests to the same individual. One of the 11 participating laboratories only shared patient identifiable information on diagnostic laboratory testing, excluding reference testing which would have come from outside the NHS trust. Demographic information was enhanced through linkage to established healthcare datasets.

A database of individuals tested for HDV was established, held and managed centrally at UKHSA. For each individual who tested HDV‐RNA positive, an NHS Trust was identified through the clinical service/setting of test reported by the laboratory. Hepatology or infectious diseases services at trusts that had 10 or more patients mapped to them were asked to submit data on their patients; including demographics (ethnicity, country of birth, primary language spoken), transient elastography (TE) assessments, clinical evidence of cirrhosis, HCC and/or transplant, information on HBV and/or HDV treatment, latest laboratory results for HBV and HDV, latest liver function test results (ALT) and HIV status. Those completing this data were also asked to note if the patient was not known to or no longer attending the clinic, or to add any additional patients attending at their clinic. Lists of patients were sent to each trust and the additional requested data was reported by completing pre‐defined fields within an excel spreadsheet or via entry into the patient management tool ‘Hepcare’ provided by BCB medical [10].

### 2.1 Data Linkage

Individuals tested for HDV were linked to NHS hospital episode statistics (HES) for inpatient stays and day cases (hospital admissions) for end stage liver disease (ESLD), hepatocellular carcinoma (HCC), Office for National Statistics (ONS) death registrations and NHS Blood and Transplant (NHSBT) liver transplant registry for liver transplant registrations and transplants. Linkage was undertaken using NHS Number. ESLD and HCC were identified using the International Classification of Diseases version 10 (ICD‐10, see Table 1), as were causes of death.

**TABLE 1:** ICD‐10 groupings.

Disease grouping	ICD‐10 codes
End‐stage liver disease (ESLD)	I850, I983, K704, K720, K721, K729, K767, R18
Hepatocellular carcinoma	C22.0

Recent postcode of residence was mapped by linking to the NHS spine personal demographics service (PDS), a national master database of all NHS patients in England, Wales and the Isle of Man, holding basic patient demographic details such as name, address, NHS number and registered GP. PDS was also used to enhance any missing NHS numbers and date of death. HES was used to enhance ethnicity.

Ethnicity was categorised into six groups:

- White British.
- Any other White ethnicity including White Irish.
- Asian including Chinese, Bangladeshi (Asian or Asian British), Pakistani (Asian or Asian British), Indian (Asian or Asian British), Any other Asian background.
- Black including African (Black or Black British), Caribbean (Black or Black British), Any other Black background.
- Mixed including any other Mixed background, White and Asian (Mixed), White and Black African (Mixed), White and Black Caribbean (Mixed).
- Other including any other ethnic group.

### 2.2 Testing Among Those Newly Diagnosed With HBV

First HBV diagnoses were obtained from routine laboratory reports, defined as the detection of hepatitis B surface antigen (HBsAg), submitted by NHS or private laboratories in England to UKHSA through surveillance forms or electronically since 1990, becoming mandatory in 2010. The first reported diagnosis in England for an individual is noted as a new diagnosis. Individuals newly diagnosed with CHB between 2011 and 2021 were collated and linked to the HDV testing data to estimate testing coverage. Region of diagnosis is mapped using a hierarchy of (i) postcode of residence, (ii) postcode of the individual's GP (both identified through linking with the NHS spine), or iii. postcode of the laboratory reporting the diagnosis. The laboratory postcode is more likely to be used where there are minimal patient identifiers for linkage.

## 3 Statistical Analysis

Data were prepared in SQL, and statistical analyses were performed using STATA version 15 (STAT Corp., College Station, Tx). The population tested for and positive for HDV was examined using descriptive analysis. Proportions were presented where data were available and compared using the chi‐squared test. A *p*‐value of < 0.05 was considered statistically significant. Multivariate models identified factors associated with having evidence of an HDV test among those newly diagnosed with CHB.

## 4 Results

**FIGURE 1:**
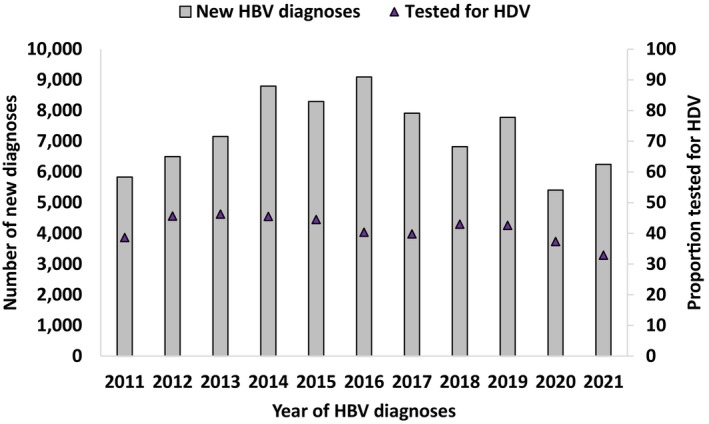
New diagnoses of hepatitis B in England by year and the proportion linked to a hepatitis D test.

Between 2011 and 2021, 97,009 individuals were newly diagnosed with CHB in England and reported to UKHSA (Figure 1). During the same time period, 55,392 individuals were tested for HDV (either antibody and/or RNA) via the 11 laboratories undertaking HDV testing in England, with an average number of 10,000 tests per year, and an average of 7000 individuals tested per year. Of the 97,009 new CHB diagnoses, 79,864 (82.3%) had identifiers to enable linkage to an HDV test, of whom 33,388 (41.8%) were linked to an HDV test, at a rate which remained stable over time between 2011 and 2019, decreasing in 2020 and 2021 (Figure 1). Declines observed in 2020 and 2021 are likely to reflect the impact of the SARS‐CoV‐2 pandemic on service provision.

**FIGURE 2:**
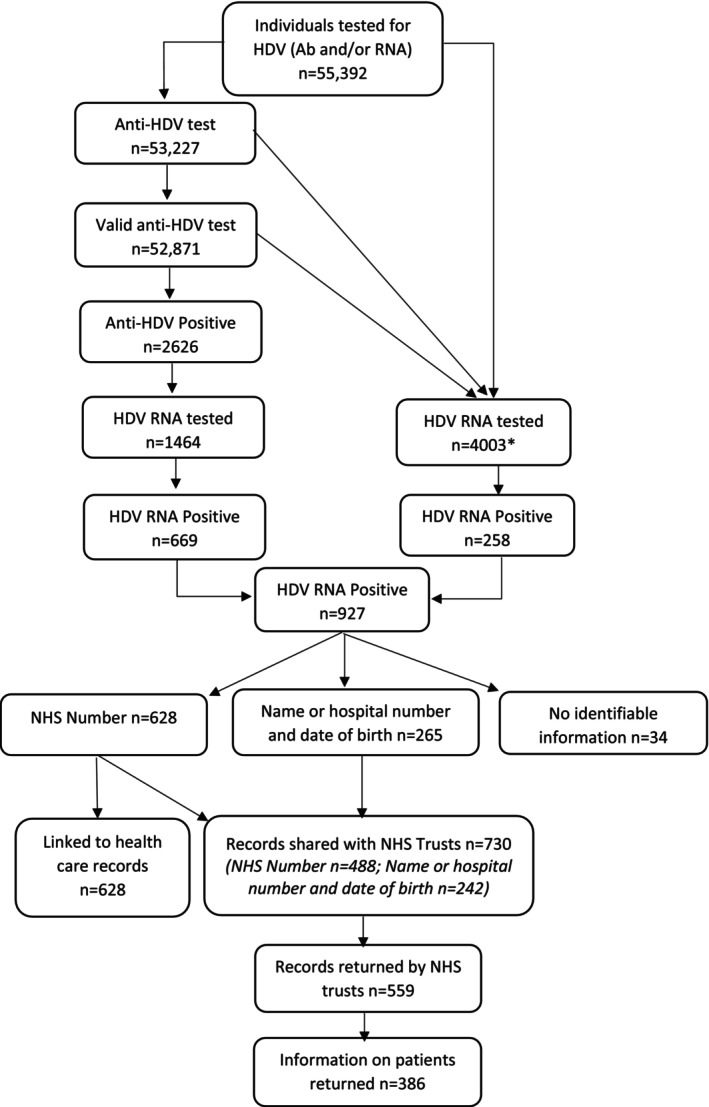
Hepatitis D testing undertaken by 11 laboratories within the UK between 2011 and 2021. * Includes 1852 without a positive anti‐HDV result and 2150 with no anti‐HDV result.

Of the 55,392 individuals tested for HDV, 53,227 individuals were tested for anti‐HDV, 52,871 had a valid result, and 2626 (5.0%) were anti‐HDV positive (Figure 2). Of those who were anti‐HDV positive, 1464 (55.8%) had a subsequent RNA test, and 669 (45.7%) were RNA positive. Median time between an HDV‐RNA test and anti‐HDV test was 0 days inter quartile range (IQR): 0–112 days, indicative of reflex testing on the same sample. 4003 individuals had an HDV‐RNA test with no record of an anti‐HDV positive test; among these, 258 (6.4%) were RNA positive. Overall, 5467 individuals had an HDV‐RNA test (with or without evidence of prior antibody testing), of which 17.0% (927) were HDV‐RNA positive. Two individuals who were HDV‐RNA positive were anti‐HDV negative at the time of testing.

To assess the burden of disease from HDV, identifiers for 78.7% (730/927) of those who were ever HDV‐RNA positive were shared with England NHS trusts, of which 76.5% (559/730) were returned by NHS trusts, and 69.1% (386/559) were known to the clinic and/or had data returned on them. Among those who were not shared with an NHS trust or did not have information returned on them, 70% had an NHS number for linking to established datasets (HES, ONS deaths, NHSBT).

### 4.1 Demographic Characteristics of those Tested and Anti‐HDV and/or RNA Positive

Males accounted for 55% of individuals tested for anti‐HDV and/or HDV‐RNA (Table 2); with no difference in anti‐HDV positivity between males and females (4.9% and 4.8% respectively; *p* = 0.89), whereas RNA positivity was higher in males compared to females (18.1% vs. 13.2% respectively; *p* < 0.001). Median age for individuals who were anti‐HDV tested was 38 years (IQR 30–48 years), and 33 years (IQR: 26–43 years) for individuals anti‐HDV positive. For individuals tested for HDV‐RNA median age was 39 years (IQR: 31–49) and 36 years (IQR: 29–44 years) among individuals RNA positive. There was little difference in median age between males and females regardless of testing or positivity.

**TABLE 2:** Demographics of individuals tested for hepatitis D in the UK between 2011 and 2021 by test type.

	Anti‐HDV test		Anti‐HDV positive		*p*	HDV RNA test regardless of Ab test		HDV RNA positive		*p*	HDV RNA test if anti‐HDV positive		*p*	HDV RNA positive		*p*
	*n*	%	*n*	%		*n*	%	*n*	%		*n*	%		*n*	%	
Total	52,871		2626	5.0		5467		927	17.0		1464	55.8		669	45.7	
Median age at test (years)	38 years (30–48 years)		33 years (26–43 years)			39 years (31–49 years)		36 years (29–44 years)			38 years (31–46 years)			35 years (29–44 years)		
Sex																
Male	27,339	55.2	1325	4.8	0.89	2435	55.4	441	18.1	< 0.001	734	55.4	0.87	382	52.0	< 0.001
Female	22,165	44.8	1068	4.8		1962	44.6	258	13.1		578	54.1		225	38.9	
Not reported	3367		233			1070		228			152			62	40.8	
Ethnicity																
White British	4635	16.04	128	2.8	< 0.001	426	13.9	41	9.6	< 0.001	75	58.6	< 0.001	36	48.0	< 0.001
White other	5477	18.96	452	8.3		737	24.0	260	35.3		320	70.8		225	70.3	
Asian or Asian British	8814	30.5	413	4.7		832	27.1	78	9.4		232	56.2		59	25.4	
Black or Black British	7628	26.4	490	6.4		809	26.3	171	21.1		311	63.5		150	48.2	
Mixed ethnicity	1031	3.57	57	5.5		119	3.9	16	13.4		36	63.2		15	41.7	
Other ethnicity	1309	4.53	95	7.3		153	5.0	41	26.8		52	54.7		36	69.2	
Not known	23,977		991			2391		320			438					
Region of current residence																
East Midlands	2462	6.59	86	3.5	< 0.001	231	6.53	28	12.1	< 0.001	50	58.1	< 0.001	26	52.0	0.002
East of England	3815	10.2	151	4.0		377	10.65	46	12.2		81	53.6		39	48.1	
London	11,826	31.63	714	6.0		935	26.42	222	23.7		357	50.0		179	50.1	
North East	1123	3	32	2.8		43	1.22	9	20.9		21	65.6		9	42.9	
North West	5702	15.25	249	4.4		466	13.17	61	13.1		194	77.9		58	29.9	
South East	4669	12.49	177	3.8		394	11.13	59	15.0		113	63.8		56	49.6	
South West	1630	4.36	59	3.6		442	12.49	24	5.4		36	61.0		21	58.3	
West Midlands	2220	5.94	117	5.3		203	5.74	36	17.7		70	59.8		33	47.1	
Yorkshire and the Humber	2995	8.01	125	4.2		262	7.4	48	18.3		90	72.0		43	47.8	
Wales	904	2.42	37	4.1		110	3.11	9	8.2		15	40.5		8	53.3	
Scotland	41	0.11	27	65.9		76	2.15	64	84.2		25	92.6		13	52.0	
Not Known	15,484		852			1928		321			412			184		

**FIGURE 3:**
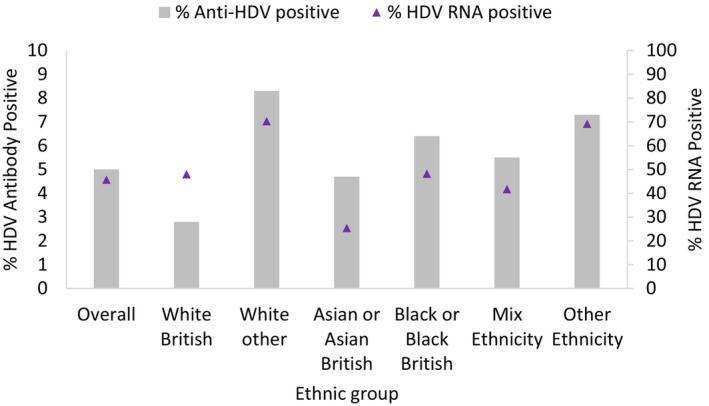
Anti‐HDV positivity and subsequent RNA positivity by ethnicity for the UK between 2011 and 2021.

Where ethnicity was available (55% of individuals tested for anti‐HDV (29,124/53,227) and 56% (3076/5467) among individuals HDV‐RNA tested), most individuals tested were of ethnic groups other than White British (84% and 86% among anti‐HDV and HDV‐RNA respectively). Anti‐HDV positivity was highest in individuals of any other white ethnicity (inc. white Irish) (8.3%), followed by any other ethnicity (7.3%), and black ethnicity (6.4%), with HDV‐RNA positivity highest in those of any other white ethnicity (inc. white Irish) (70.3%), followed by other ethnicity (69.2%), and black ethnicity (48.2%) (Figure 3). Demographic trends over time are presented in the Supporting Information (Section S2; Figure S1–S6). London had the highest number of anti‐HDV and/or HDV‐RNA tests and the highest positivity with 6.0% anti‐HDV positivity and 26.4% HDV‐RNA positivity (regardless of an antibody test). The South West had the highest RNA positivity among individuals' antibody positive (58.3%).

When comparing individuals who were anti‐HDV positive and anti‐HDV negative, there was little difference in the distribution of sex, age at diagnosis, region of diagnosis.

**FIGURE 4:**
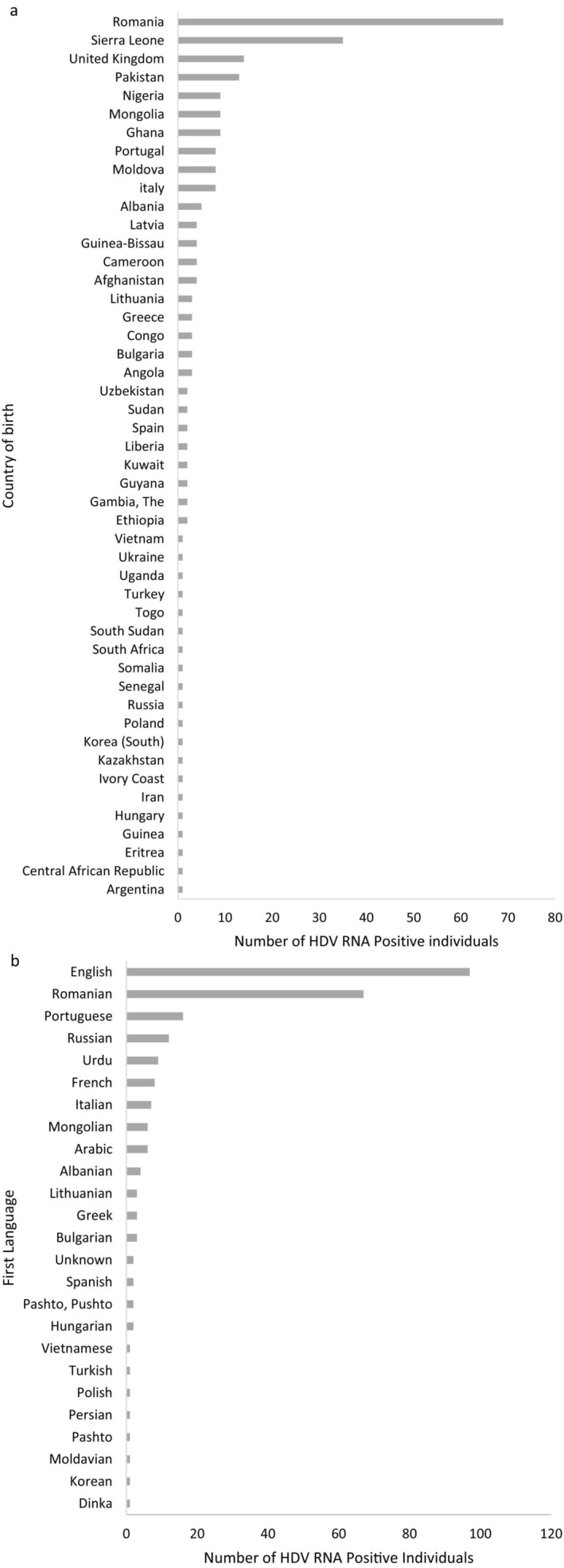
(a) Number of individuals HDV RNA positive by country of birth in England, between 2011 and 2021. (b) Reported first language for individuals RNA positive in the UK between 2011 and 2021.

Among 386 individuals' RNA positive where data was returned by NHS trusts, 265 (68.7%) had country of birth (COB) reported of which 94.3% (250/265) were born outside the UK. Figure 4a shows the distribution of COB; Romania was the highest reported COB (26.0%; 69), followed by Sierra Leone (13.2%; 35). Where primary language was reported (261; 67.6%), 24 different languages were reported with Romanian the most common, followed by Portuguese and Russian (Figure 4b).

### 4.2 HDV Testing Among Those Newly Diagnosed With CHB

Comparing individuals who were linked to a HDV test with individuals who were not, women with CHB were more likely to be tested for HDV than men (44% vs. 41% respectively; *p* < 0.001). Individuals aged 25–34 years were more likely to be tested for HDV than all other age groups (*p* < 0.001) and individuals of White British ethnicity were less likely to be tested than all other ethnic groups (*p* < 0.001). There was regional variation (*p* < 0.001) with individuals diagnosed in the North East with the highest proportion tested for HDV (62%) and the West Midlands with the lowest (23%). In an adjusted model, age, region of diagnosis and ethnicity remained independent factors for testing for HDV (Table 3).

**TABLE 3:** Demographics of individuals newly diagnosed with hepatitis B and linked to a hepatitis D test in England between 2011 and 2021.

	HBV diagnosis		HDV test						
	*n*	%	*n*	%	*p*	aOR	95% CI		
New diagnoses	97,009								
With identifiers	79,864	82.3	33,388	41.8					
Median age	37 years (30–48 years)		36 years (29–46 years)						
Age group									
< 25	7126	8.9	3079	43.2		0.95	0.89	1.01	< 0.001
25–34	25,398	31.8	11,617	45.7		1			
35–44	21,212	26.6	9327	44.0		0.93	0.90	0.97	
45–54	13,252	16.6	5278	40.0		0.83	0.79	0.87	
55–64	7011	8.8	2509	36.8		0.71	0.67	0.76	
≥ 65	5865	7.3	1578	26.9		0.50	0.47	0.54	
Sex									
Male	45,055	56.8	18,314	40.7	< 0.001	1			0.12
Female	34,205	43.2	14,937	43.7		0.97	0.43	1.01	
Not reported	604		137						
Ethnicity									
White British	14,476	22.7	5277	36.5	< 0.001	1			< 0.001
White other	12,215	19.1	6422	52.6		1.81	1.72	1.91	
Asian or Asian British	16,341	25.6	8733	53.4		1.98	1.89	2.08	
Black or Black British	15,818	24.8	8377	53.0		1.95	1.86	2.04	
Mixed ethnicity	1901	3.0	1029	54.1		1.98	1.79	2.18	
Other ethnicity	3168	5.0	1634	51.6		1.81	1.67	1.96	
Not known	15,945		1916						
Region of diagnosis									
East Midlands	4031	5.1	1941	48.2		1.69	1.56	1.83	
East of England	5387	6.8	2863	53.2		1.64	1.54	1.75	
London	35,868	45.1	14,446	40.3		1			< 0.001
North East	1611	2.0	1003	62.3		2.49	2.22	2.80	
North West	8138	10.2	4124	50.7		1.36	1.29	1.43	
South East	7466	9.4	3419	45.8		1.47	1.39	1.56	
South West	3903	4.9	1303	33.4		0.80	0.74	0.86	
West Midlands	6696	8.4	1559	23.3		0.45	0.42	0.48	
Yorkshire and the Humber	6459	8.1	2688	41.6		1.16	1.09	1.23	
Not known	305		42						

### 4.3 Recent Laboratory Tests

Among 386 RNA positive individuals where data was returned by NHS trusts, 328 recorded the individual's latest HDV laboratory results with 97 (29.6%) recorded as HDV‐RNA negative. Forty‐eight of these individuals had received treatment for HDV, 42 had not. There was no difference in recent RNA status among individuals with no evidence of treatment by sex and age; however, ethnicity was associated with a change in RNA status (*p* < 0.001). Individuals of black or Asian ethnicity had a higher proportion RNA negative (64.3% (9/14) and 38.8% (19/49) respectively). Among individuals with evidence of treatment, there was no difference in RNA clearance by age and sex; however, individuals of black ethnicity had a higher proportion of clearance, 65.3% (32/49).

Where test information was available for both HBeAg and anti‐HBe status (82%; 316/386), 254 (80%) were HBeAg negative and anti‐HBe positive, 23 (7%) were negative for both markers, 7 (2%) were positive for both markers, and 32 (10%) were HBeAg positive and anti‐HBe negative.

### 4.4 Clinical Characteristics

Clinical information on HDV‐RNA positive individuals as supplied by NHS trusts and/or through data linkage is summarised in Table 4.

**TABLE 4:** Clinical data reported by NHS trusts in England and/or through data linkage for individuals HDV‐RNA positive between 2011 and 2021.

RNA Positive with information returned from NHS trusts			RNA Positive with NHS Number for linkage			Either NHS no and/or information returned		
	*n*	%		n	%		n	%
Total RNA positive	927			927			927	
Total of those RNA positive	386			628			764	
*Is the patient known to be cirrhotic*								
Yes	167	47.7						
No	183	52.3						
Not reported	36							
*Evidence of decompensation*								
Yes^a^	59	26.5						
No	164	73.5						
Not reported	163							
			*Linked to HES ESLD diagnosis (inpatient)*					
			Yes	87	13.9			
			No	541	86.1			
*Evidence of HCC*			*Linked to HES HCC diagnosis (inpatient)*			*HCC*		
Yes	30	9.1	Yes	28	4.5	Yes	47	6.2
No	298	90.9	No	600	95.5	No	717	93.8
Not reported	35							
*Has the patient had a liver transplant*			*Linked to NHSBT liver transplant registry*			*Transplant*		
Yes	41	15.5	Yes	45	7.2	Yes	70	9.2
No	224	84.5	No	583	92.8	No	694	90.8
Not reported/not applicable	121							
			*Linked to ONS death registrations*					
			Yes	60	9.6			
			No	568	90.4			
*Median ALT (IU/L)*	49 (29–86)							
Median liver stiffness measurement (kPa)	7.8 kPa (5.6–12.5)							
Median CAP (dB/m)	220 dB/m (188–255)							

^a^ Two not due to cirrhosis.

Among this group, 48.8% (177/363) had evidence of ever being treated for HDV, the majority of whom (159/177, 89.8%) were prescribed interferon or peg interferon; four had been prescribed bulevirtide. 62.9% (232/369) had evidence of ever being treated for HBV, the majority (59%; 138) with tenofovir, followed by entecavir (28.4%; 66).

Using data returned by the NHS trusts on those HDV‐RNA positive (*n* = 386), where reported 48% (167/350) were reported as cirrhotic, and 26% (59/223) had evidence of decompensation. Median liver stiffness of individuals was 7.8 kPa (5.6–12.5), median ALT was 49 (29–86), and median CAP score of 220 dB/m (188–255) and 9% (30/328) of individuals had a HCC diagnosis. An additional 17 HCC diagnoses were identified through linking to inpatient records resulting in 6% (47/764) with diagnosed HCC. 15% (41/265) of individuals were reported by the NHS trust as being assessed for or having received a transplant falling to 9% (70/764) with additional linkage to the NHS Blood and Transplant (NHSBT) registry. Among all HDV‐RNA positive individuals, linking to death registrations identified that 9.6% (60) had died, most died of either liver cancer (27.8%) or decompensated chronic liver disease (30.6%).

### 4.5 Factors Associated With Clinical Status

Increased age at HDV‐RNA diagnosis was associated with evidence of cirrhosis (*p* = 0.006), assessment for or having a liver transplant (*p* = 0.007), and being diagnosed with HCC (*p* < 0.001). Ethnic group was associated with having cirrhosis (*p* = 0.04), with individuals of other white background (55.1%) and individuals of Asian ethnicity (41.3%) with the highest proportion and individuals of Black ethnicity with the lowest (25.6%). Ethnic group was also associated with being assessed for or having a liver transplant (*p* = 0.018), with the highest proportion among individuals of White British ethnicity (17.7%) followed by Asian ethnicity (15.2%) and individuals of White Other ethnicity with the lowest proportion (12.4%).

## 5 Discussion

Our findings show variability in testing for HDV in England, with less than half of individuals newly diagnosed with CHB in England between 2011 and 2021 being screened for HDV, and only 56% of those testing anti‐HDV positive having an RNA test. Where testing did occur anti‐HDV positivity was 5.0%, and among those HDV‐RNA tested, regardless of a previous Ab test, positivity was 14.6%. RNA positivity was higher (45.7%) where there was a previous antibody positive result available. The distribution of individuals positive for HDV reflects the epidemiology of individuals with CHB in England, with positivity highest among ethnic minorities, and in areas where the underlying population is more diverse. These findings may also reflect testing biases with HDV testing offered proactively in individuals from highly endemic countries. However, these data show that there was no difference in the demographics of individuals living with diagnosed CHB who were positive vs. negative for evidence of HDV infection, consistent with the recommendation by EASL and NICE to test all individuals with CHB and not risk based testing as recommended by AASLD. These data have identified gaps and inefficiencies in testing pathways for HDV, and analysis of clinical characteristics of HDV‐RNA positive individuals suggests the enhanced pathogenicity of HBV/HDV co‐infection.

The rate of HDV testing among individuals known to have CHB was disappointingly low. In those tested, anti‐HDV positivity was similar to other studies ranging from 2% to 10.6% [1, 6, 7, 11, 12, 13, 14]. HDV‐RNA positivity among those known to be antibody positive was also similar to prior studies ranging from 46.6% to 62.4% [1, 12, 15, 16]. However, the positivity among all individuals tested for HDV‐RNA was much lower, likely associated with RNA testing in the absence of an anti‐HDV result. A more efficient testing pathway would be reflex RNA testing only if anti‐HDV is positive. Variations in HDV‐RNA testing may have been due to a historical lack of a commercial HDV‐RNA assay and the absence of standardisation. Similar challenges have been reported by Rodriguez‐Tajes et al., where in Spain two out of five centres did not have an HDV‐RNA qualitative assay available, with 20% of tests sent to another laboratory for testing [15]. Whilst there are commercial assays now available within the UK, none are CE‐IVD marked and further work is needed to evaluate their comparability. Another possibility is that individuals with an RNA test in the absence of an antibody test may have been initially diagnosed prior to the study period when the antibody would have been conducted, and the RNA tests within the study represent appropriate monitoring and diagnosed patients.

There is consistent evidence that HBV/HDV coinfection is associated with severe disease progression, with an increased risk of developing cirrhosis and HCC [12, 14, 16, 17, 18, 19]. Wranke et al. recently published findings suggesting that whilst progression to cirrhosis was faster in those with HDV, progression to liver‐related end points was no different between HBV mono‐infected individuals and HBV/HDV coinfected individuals with cirrhosis [13]. In the absence of a HBV mono‐infected comparator we are unable to present statistical comparisons, however, among our cohort of HDV positive individuals the proportions with disease progression were high, with two out of every five individuals having evidence of cirrhosis, and a third reported as having decompensated cirrhosis. Whilst previously there was little difference in prognosis associated with treatment, the recent availability of bulevirtide is likely to change the treatment landscape, with reduction in HDV viraemia levels and normalisation of ALT levels likely to result in better outcomes for individuals [20, 21]. However, to benefit from treatment a diagnosis is required. With less than half of those newly diagnosed with CHB in England receiving a HDV test this is a significant limitation in the assessment and referral of patients. Significant efforts are needed to ensure all individuals living with CHB are screened for HDV to have the opportunity for treatment. Similar to testing pathways for individuals screened for HCV, testing for HDV should occur using the same sample as a positive HBsAg result, and a test for active infection in those samples found to be anti‐HDV positive (i.e., RNA testing) should occur on the same sample the antibody test was conducted on, known as reflex testing. The use of reflex testing in Spain, established in 2021, resulted in a five‐fold increase in the number of HDV diagnoses [22], whilst modelling in the US suggests that double reflex testing on the HBsAg positive sample, followed by the anti‐HDV positive sample, results in earlier detection, increasing numbers diagnosed and treated, thereby reducing disease progression and associated morbidity and mortality [23]. Further work is required to understand barriers to double reflex testing in the UK, barriers are likely to include the cost associated with reflex testing for those HBsAg positive, availability of sample volumes after testing for all HBV markers, and a lack of awareness of and feasibility of HDV testing. These themes align with a recent survey of healthcare professionals in the UK, alongside staff forgetting to test [24]. However, the cost of reflex testing should be weighed against the possible high economic impact of individuals with undiagnosed HDV and the associated disease progression as outlined in a Spanish study [25].

Some 30% of those who were HDV‐RNA positive had a recent RNA negative result suggesting clearance of HDV‐RNA. This was a similar proportion to that reported from an Italian study with approximately 20%–30% of patients with intermittent or persistently undetectable HDV‐RNA [14, 26]. Data from Spain evaluating changes in serum samples among people with a detectable HDV‐RNA concluded that one in four untreated patients showed an RNA decline, with undetectable levels reached in 20% [27]. In our data, we saw a significant difference by ethnic group, with those of Black or Asian ethnic origin more likely to clear HDV‐RNA in individuals with no evidence of treatment, and among Black ethnic origin in individuals with evidence of treatment. Further work is needed to understand factors associated with clearance without treatment, including differences by genotype and RNA assay used [28].

Only half of individuals' RNA positive had evidence of ever being treated, predominately with interferon, and at the time of this study only a small number had the opportunity to receive bulevirtide due to its recent availability. Ongoing monitoring is needed to understand the impact of new therapies for HDV on both patient outcomes and on clinical capacity. Frequent monitoring by RNA testing of patients receiving interferon or bulevirtide is required, and long‐term data on the occurrence of cirrhosis and HCC will be needed in order to assess clinical impact.

Recent prevalence estimates for England suggest that nearly 270,000 individuals are living with CHB, with the majority belonging to ethnic minority groups [29]. The authors discuss the unmet need and associated equity issues among this population compared to UK‐born patients, with barriers associated with testing and treatment including language, stigma and poor knowledge and understanding of viral hepatitis. Not unexpectedly, our data on HDV mirrors that of HBV. Among those with a positive HDV‐RNA, 24 different languages were noted and less than half indicated English as their primary language. This was similar to that reported by Jack et al. [24], with language and communication difficulties a barrier for access to and long‐term maintenance of clinic attendance. If we are to address the challenges in the management of individuals living with CHB (and HDV), we must consider the needs of the individual, explore alternative patient pathways, and methods by which to communicate with patients to ensure long‐term engagement with clinical care.

Our study is limited by the fact that we were not able to access data from all testing laboratories, and HDV testing may have occurred in laboratories not included in this study, thereby resulting in an underestimate of the number of individuals within the testing pathway and differences in the individual characteristics. Patient identifiable information needed for deduplication and identification of the same patients being tested in more than one laboratory was complete for most individuals; however, where data was missing, this could lead to an underestimate of the number of individuals tested for both anti‐HDV and HDV‐RNA.

## 6 Conclusion

HDV testing in the UK is sub‐optimal, both HDV testing among those HBsAg positive individuals and HDV‐RNA testing among those found to be anti‐HDV positive. Whilst the introduction of ‘reflex’ testing would benefit HDV testing pathways, this needs to be considered alongside the additional costs and the need for appropriate resources for hepatitis clinical services. There is considerable diversity among HDV‐RNA positive individuals and evidence suggesting that coinfection with HDV is associated with increased disease severity. Considerable work is needed to improve the patient care pathway to ensure patients are appropriately diagnosed, linked and retained in care, with adequate access to treatment and to strengthen guidance around the implementation and optimisation of testing.

## Author Contributions

R.S., S.I., W.I. and P.T.K. submitted the grant application. R.S., R.M., R.R., R.J. and K.J. collated the data submitted by laboratories and NHS acute trusts. A.R., A.E., A.U., A.K., A.C., A.L., A.C., A.E., A.B., B.S., C.L., D.B., D.F., D.M., D.F., E.N., E.B., E.S., E.P., I.C., F.J.V., H.M., J.C., J.S., K.A., K.B., K.A., K.D., L.R., M.B., H.Z., L.R., M.M., M. Ankcorn, M. Aldersley, M.P., M.L., M.F, M. Wright, M.C., M. Wiselka, N.E., P.R., R.G., R.H., R.B., R.J., R.A., R.S., R.C., S.A., S.M., S.R., S.V., T.L., V.S., W.G., P.T.K. and W.L.I. all submitted data from their sites, and provided insights to the results and discussion. R.S. undertook the analysis and had access to the complete dataset. R.S., S.I., W.L.I. and P.T.K. drafted the manuscript. D.L., M.D. and S.M. reviewed and provided input into the manuscript. All authors reviewed and approved the manuscript submission.

## Funding

This work was supported by Gilead Sciences, IN‐UK‐980‐6339.

## Ethics Statement

The data were collected for the surveillance of viral hepatitis (A‐E) including linkage to other healthcare data sets and is covered by Health Service (Control of Patient Information) Regulations 2002 (regulation 3) and under Section 251 of the NHS Act 2006. UKHSA's Caldicott Advisory Panel approval was obtained for managing and processing data related to the surveillance of viral hepatitis (A‐E) and the collection of clinical data was reviewed by the UKHSA Research Ethics and Governance Group (REGG) and approval was given that these data are viewed as part of surveillance activities (NR0292).

## Conflicts of Interest

The authors declare no conflicts of interest.

## Supporting information


**Data S1:** jvh70205‐sup‐0001‐Supinfo.docx.
**Section S1**. HDV RNA assays used by the laboratories within England.
**Section S2**. Demographic trends over time.
**Figure S1:** Number of individuals HDV antibody positive by sex and year of diagnosis for the UK between 2011and 2021.
**Figure S2:** Age distribution of individuals HDV antibody positives by year of diagnosis for the UK between 2011 and 2021.
**Figure S3:** Proportion of individuals HDV antibody positive by ethnic group for the UK between 2011 and 2021.
**Figure S4:** Number of individuals HDV RNA positive by sex and year of diagnosis for the UK between 2011and 2021.
**Figure S5:** Age distribution of individuals HDV RNA positives by year of diagnosis for the UK between 2011 and 2021.
**Figure S6:** Proportion of individuals HDV RNA positive by ethnic group for the UK between 2011 and 2021.

## Data Availability

The data that support the findings of this study are available on request from the corresponding author. The data are not publicly available due to privacy or ethical restrictions.
