# Supplementary material for: Characterising Hepatitis D Virus in the UK From 2011 to 2021
Source: J Viral Hepat. 2026 Aug 9;33(9):e70205. doi: 10.1111/jvh.70205 (PMC13454316; doi:10.1111/jvh.70205)
Supplement: Supplementary file 1 [file JVH-33-0-s001.docx]

**Supplementary materials**

**Section 1: HDV RNA assays used by the laboratories within England**

***Micropath LtD:***

2011 to 16/06/2015 - Nested -RT-PCR based on primers described in ‘Etiological Considerations of Fulminant Non-A, Non-B viral Hepatitis in Japan: Analyses by Nucleic Acid Amplification Method. Fukai *et al*., 1998. Journal of Infectious Disease. Vol 178. p 325 – 333.

17/06/2015 until 19/01/2021 - Ferns R, Nastouli E, Garson J (2012) Quantitation of hepatitis delta virus using a single-step internally controlled real-time RT-qPCR and a full-length genomic RNA calibration standard *J Virol Met* 179:189-94.

20/01/2021 to present - Altona RealStar HDV RT-PCR kit 1.0.

***Kings College London:***

2011-2019 - DiaSorin ETI-AB-DELTAK-2 (P2808)

2019-2021 - Diapro Hepatitis D Virus (HDV) Antigen ELISA

HDV RNA assay is an in-house quantitative assay

***Glasgow:***

Mederacke I (2010). [Establishment of a Novel Quantitative Hepatitis D Virus (HDV) RNA Assay Using the Cobas TaqMan Platform To Study HDV RNA Kinetics - PMC](https://eur01.safelinks.protection.outlook.com/?url=https%3A%2F%2Fpmc.ncbi.nlm.nih.gov%2Farticles%2FPMC2884474%2F&data=05%7C02%7CRuth.Simmons%40ukhsa.gov.uk%7C91af7fe0c98a4617807708deb0036115%7Cee4e14994a354b2ead475f3cf9de8666%7C0%7C0%7C639141725739196597%7CUnknown%7CTWFpbGZsb3d8eyJFbXB0eU1hcGkiOnRydWUsIlYiOiIwLjAuMDAwMCIsIlAiOiJXaW4zMiIsIkFOIjoiTWFpbCIsIldUIjoyfQ%3D%3D%7C0%7C%7C%7C&sdata=m6EVeYt7jsJv1Lvt5qukP2LiQPwEIC7ANNBD0Y0cCy8%3D&reserved=0) J Clin Microbiol. 2010 Mar 29;48(6):2022–2029

***UKHSA BBVU:***

HDV RNA qual inhouse assay: 01/10/2009

HDV RNA quant inhouse assay: 21/03/2019

HDV Altona assay: 07/04/2021

***University College London:***

Ferns RB, Nastouli E, Garson JA. Quantitation of hepatitis delta virus using a single-step internally controlled real-time RT-qPCR and a full-length genomic RNA calibration standard. J Virol Methods. 2012 Jan;179(1):189-94. doi: 10.1016/j.jviromet.2011.11.001. Epub 2011 Nov 15

El Bouzidi K, Elamin W, Kranzer K, Irish DN, Ferns B, Kennedy P, Rosenberg W, Dusheiko G, Sabin CA, Smith BC, Nastouli E. Hepatitis delta virus testing, epidemiology and management: a multicentre cross-sectional study of patients in London. J Clin Virol. 2015 May;66:33-7. doi: 10.1016/j.jcv.2015.02.011. Epub 2015 Feb 20. PMID: 25866333.

***Leeds infirmary:***

Alinity M

Roche AmpliPrep

**Section 2: Demographic trends over time.**

Figure S1: Number of individuals HDV antibody positive by sex and year of diagnosis for the UK between 2011and 2021.

Figure S2: Age distribution of individuals HDV antibody positives by year of diagnosis for the UK between 2011 and 2021.

Figure S3: Proportion of individuals HDV antibody positive by ethnic group for the UK between 2011 and 2021.

Figure S4: Number of individuals HDV RNA positive by sex and year of diagnosis for the UK between 2011and 2021.

Figure S5: Age distribution of individuals HDV RNA positives by year of diagnosis for the UK between 2011 and 2021.

Figure S6: Proportion of individuals HDV RNA positive by ethnic group for the UK between 2011 and 2021.
